# Supplementary material for: Unraveling the Spatiotemporal Distribution of VPS13A in the Mouse Brain
Source: Int J Mol Sci. 2021 Dec 1;22(23):13018. doi: 10.3390/ijms222313018 (PMC8657609; doi:10.3390/ijms222313018)
Supplement: Supplementary file 1 [file ijms-22-13018-s001.zip › ijms-1461614-supplementary.pdf]

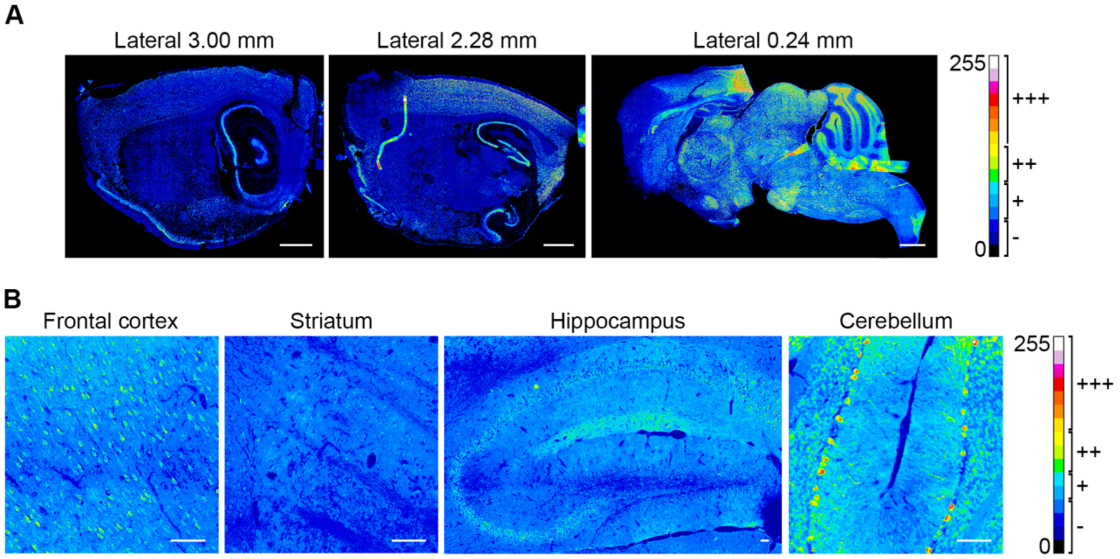

Supplementary figure S1: Representative pseudocolor images of VPS13A expression in the mouse brain. Pseudocolor images of A) mRNA labelling by FISH in sagittal sections at 3 different brain levels, and B) VPS13A immunodetection in histological sections of cerebral cortex, striatum, hippocampus and cerebellum. Color scales show the pseudocolor codes used for semiquantitative evaluation of VPS13A mRNA and protein labelling respectively. Scale bars = 1 mm for A and 50 µm for B.

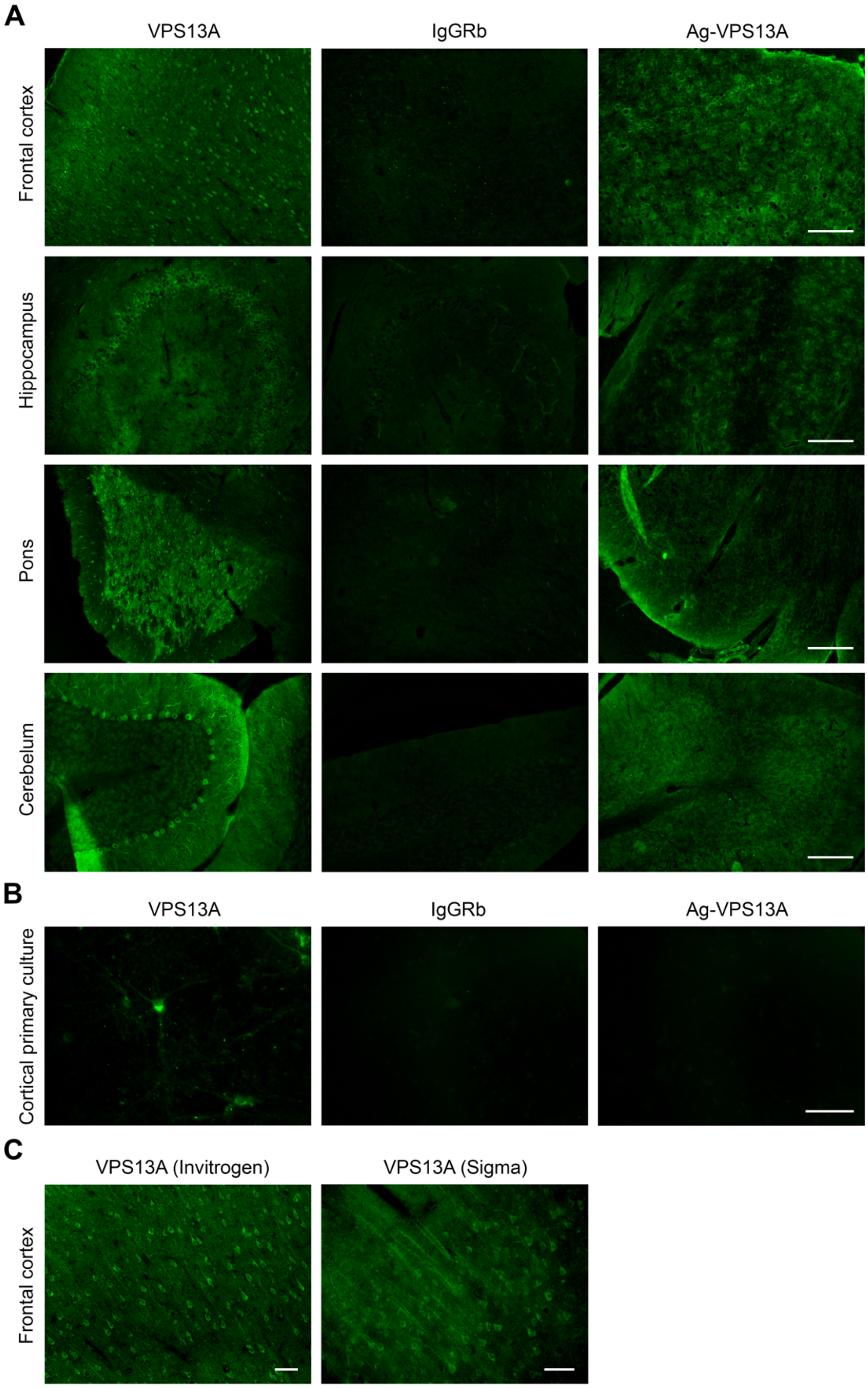

Supplementary Figure S2: The anti-VPS13A antibody is specific for immunochemistry. Incubation with rabbit IgG as primary antibody (IgGRb) and with a mixture of anti-VPS13A antibody and its antigen (Ag-VPS13A) (1:100, relative concentrations) were used as negative controls. A) Representative images of VPS13A immunostaining in sagittal section of the adult mouse brain. n = 3. Scale bar 150µm. B) Representative images of VPS13A immunostaining in cortical primary cultures. n = 3. Scale bar 50µm. C) Illustrative images of VPS13A immunelabelling in frontal cortex slices using anti-VPS13A antibodies from Invitrogen (Cat: PA5-54483) and from Sigma (Cat: HPA021662) with similar staining pattern. Scale bar 50µm

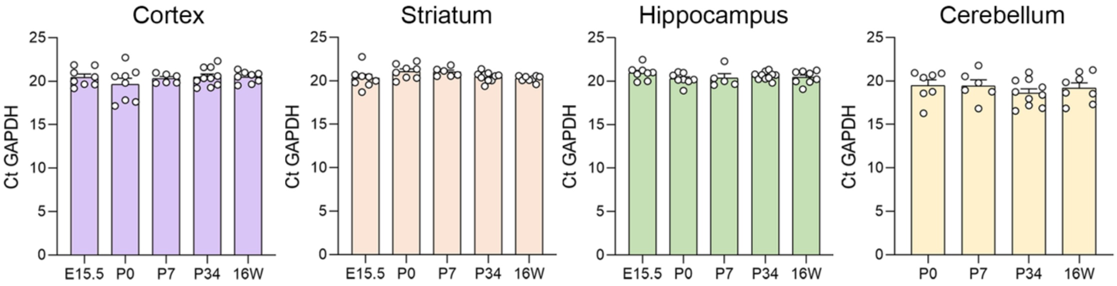

Supplementary figure S3: GAPDH expression quantified by qRT-PCR in four areas of the mouse brain. One-way ANOVA analysis of the mean qRT-PCT Ct values evidenced no time-related changes of GAPDH expression in the frontal cortex ($F_{(4, 35)}$ = 0.729; p = 0.577), striatum ($F_{(4, 35)}$ = 2.029; p = 0.111), hippocampus ($F_{(4, 34)}$ = 1.043; p = 0.399) or cerebellum ($F_{(3, 27)}$ = 0.519; p = 0.672) (n = 6-10 animals/group)

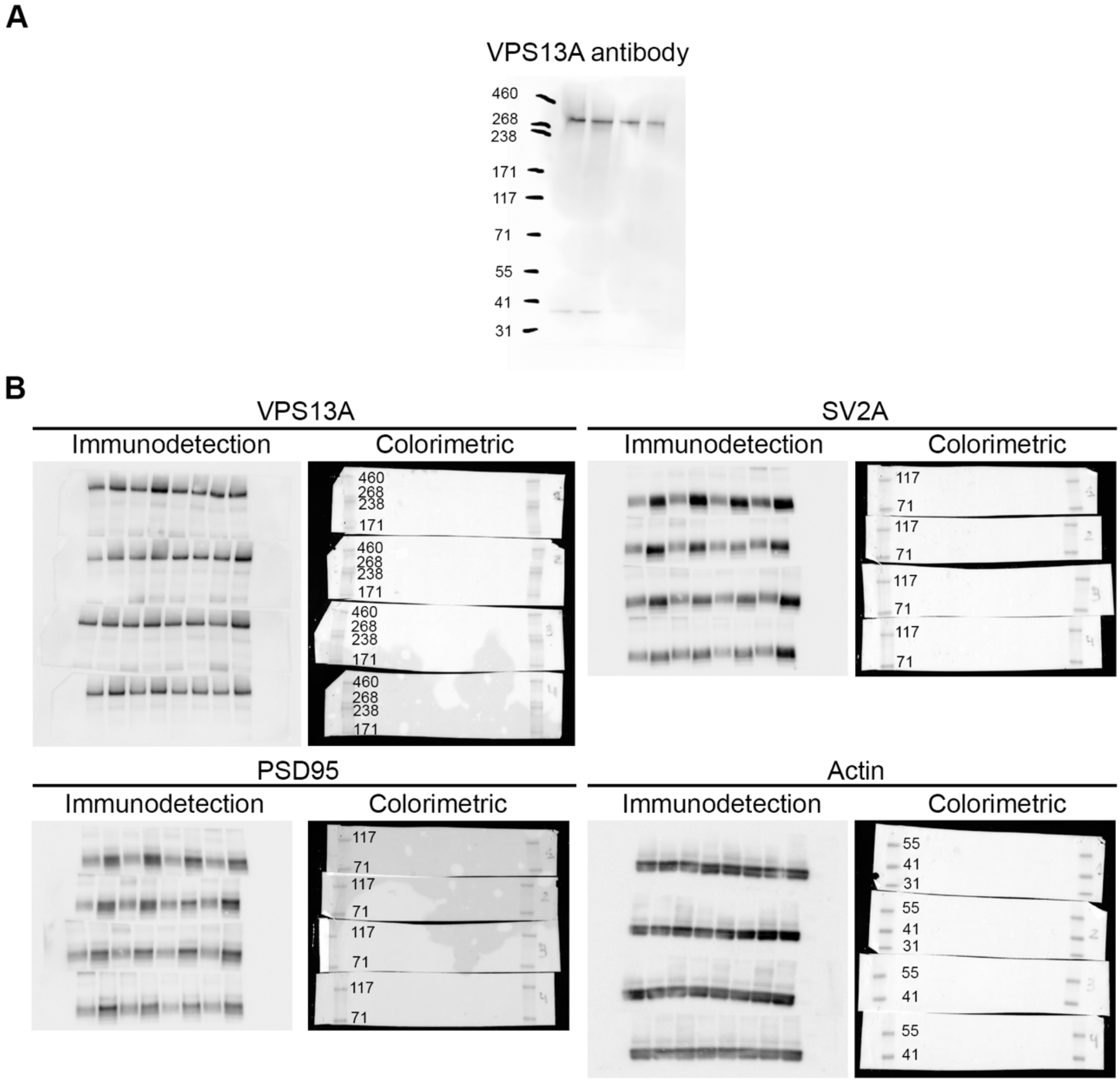

Supplementary figure S4: Pictures of the whole membranes of western blot of A) VPS13A immunoblot and (B) of figure 6

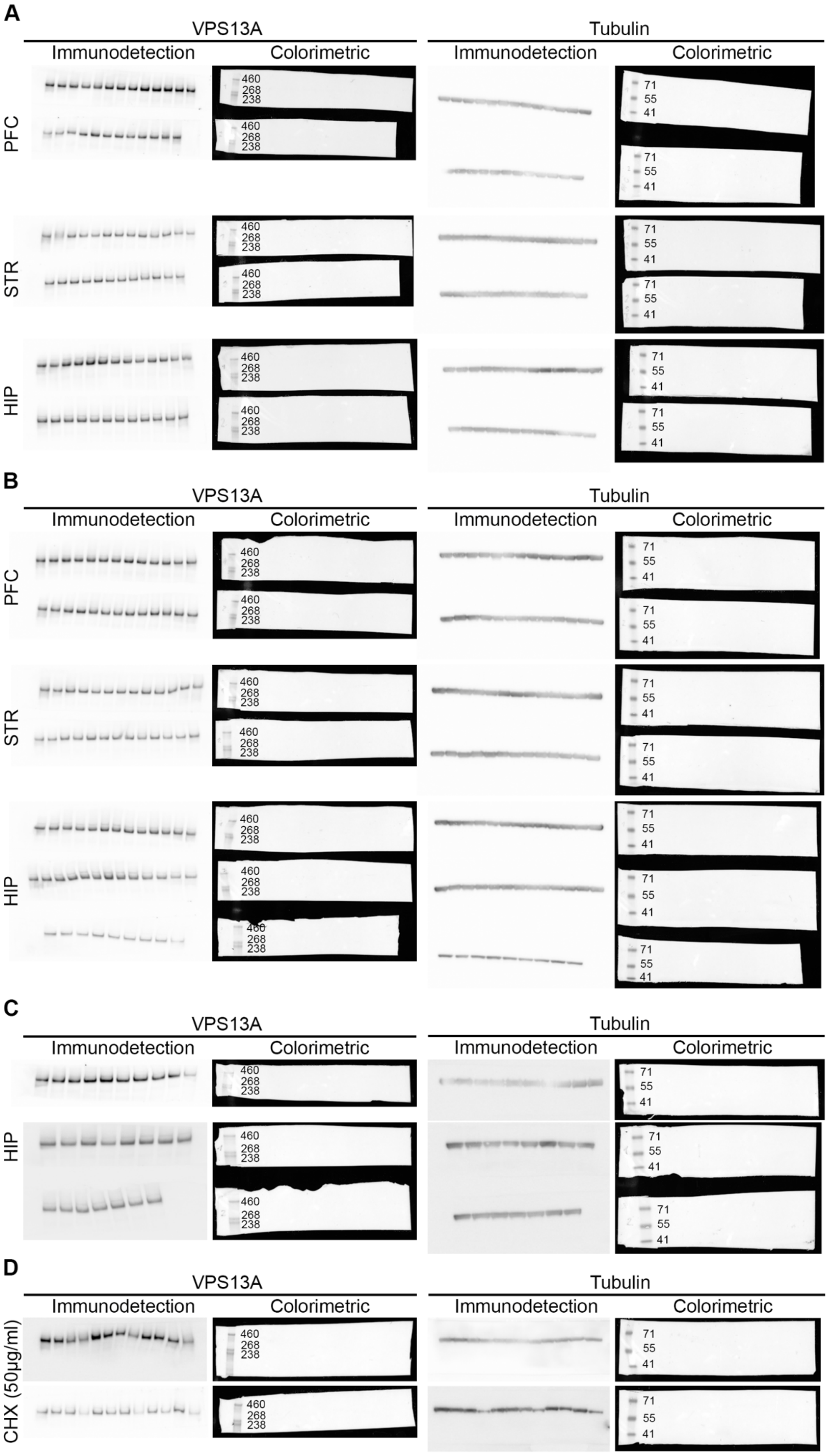

Supplementary figure S5: Pictures of the whole membranes of western blot of figure 7.
